# Updates in Endoscopic Bariatric and Metabolic Therapies

**DOI:** 10.3390/jcm12031126

**Published:** 2023-01-31

**Authors:** Hammad Qureshi, Naba Saeed, Manol Jovani

**Affiliations:** 1 Division of Hospital Medicine, Department of Internal Medicine, University of Kentucky, Lexington, KY 40536, USA; 2 Division of Gastroenterology, Department of Internal Medicine, University of Kentucky, Lexington, KY 40536, USA; 3 Division of Gastroenterology, Maimonides Medical Center, Brooklyn, NY 11219, USA

**Keywords:** endoscopic bariatric and metabolic therapies (EBMTs), obesity, weight loss

## Abstract

The rising prevalence of obesity, and related morbidity and mortality, has necessitated the development of therapeutic weight loss strategies. Lifestyle modifications alone have only yielded modest benefit, and while bariatric surgery has shown significant short- and long-term results, only a minority of eligible patients end up receiving this treatment. Endoscopic bariatric and metabolic therapies (EBMTs) are a rapidly evolving field, which provides a less invasive middle ground treatment option for weight loss. Here we discuss the efficacy, as well as short- and long-term outcomes with restrictive, malabsorptive/metabolic and aspiration endoscopic techniques, and their effects on metabolic parameters.

## 1. Introduction

With the increasing obesity epidemic, obesity-related comorbidities are on the rise including coronary artery disease (CAD), diabetes, hypertension, hyperlipidemia, non-alcoholic fatty liver disease (NAFLD), obstructive sleep apnea (OSA), gastroesophageal reflux disease (GERD) and others [1,2]. More than 40% of the adult population in the USA is obese, and in 2019 alone, nearly USD 173 billion was spent on obesity-related healthcare costs [2]. As such, obesity and its complications have been recognized as a public health crisis and concerted efforts targeting development of preventive and treatment strategies is ongoing [2,3]. Obesity is typically defined as body mass index (BMI) ≥ 30. Bariatric interventions are indicated in patients with BMI ≥ 40, or BMI 35–39.9 and one obesity-related comorbidity, or BMI 30–34 with uncontrolled type 2 diabetes or metabolic syndrome [1]. In addition, weight loss is the cornerstone of therapy for non-alcoholic fatty liver disease (NAFLD). A sustained weight loss of 7–10% TBW is needed to decrease steatohepatitis, steatosis and fibrosis; however, this is often difficult to achieve and sustain [4,5]. Thus far, lifestyle and medical therapies have been shown to yield only modest, short-term weight loss, with most studies showing loss of effect after two years of implementation. Bariatric surgery, on the other hand, is more invasive but has demonstrated more durability than medical therapies for up to 20 years [6].

Endoscopic bariatric and metabolic therapies (EBMTs) are in a rapidly evolving field and are generally considered less invasive than bariatric surgery, but more effective than medications and lifestyle interventions. These procedures meet the thresholds put forth by the American Society of Gastrointestinal Endoscopy (ASGE) taskforce: >5% total body weight loss (TBWL), >25% excess body weight loss (EBWL) and <5% risk of adverse events [7]. The EBMT procedures can largely be subdivided into restrictive, malabsorptive/metabolic and aspiration techniques depending on their target [8]. In this paper we will review the current status of endoscopic therapeutic options for obesity. 

## 2. Restrictive EBMTs

### 2.1. Intra-Gastric Balloons (IGBs)

These are space-occupying balloons that are designed to fill a large portion of the stomach, thereby delaying gastric emptying and increasing sensation of satiety, leading to reduced oral intake. They are approved for use in patients with BMI 30–34.9 and ≥1 obesity-related comorbidities who have failed medical/lifestyle interventions [9]. The Orbera, ReShape, Obalon, Transpyloric shuttle and Spatz3 are FDA approved IGBs, while the Elipse Balloon is still not FDA approved. Of these, the Obalon and the Elipse balloons are swallowable balloons, while other balloons are placed endoscopically and filled with air or fluid. Intragastric balloons can stay in place for about 4–12 months, depending on the type of balloon [6,10].

The Orbera Balloon is the most widely used IGB, approved for use by the FDA in patients with BMI 30–40 kg/m^2^. It is endoscopically placed and removed up to 6 months after placement but can be placed multiple times. A meta-analysis of 1638 patients following Orbera placement demonstrated a mean sustained EBWL of 25.44% (95% CI, 21.47–29.4) at 12 months [7]. In three randomized controlled trials (RCTs) comparing Orbera balloon placement to controls, % EBWL was 26.9% (95% CI, 15.6–38.2; *p ≤* 0.01) [7].

In a large meta-analysis that included 5668 patients, IGBs were shown to decrease waist circumference (−4.1 cm), improve diastolic blood pressure (−2.9 mm Hg), reduce A1c (−1.1%) and fasting glucose levels (−12.7 mg/dL), as well as triglyceride levels (−19 mg/dL). Mean EBWL was 31.8% and mean TBWL was 11.1%. The odds for diabetes resolution was 1.4% following IGB therapy and the serious adverse event rate was 1.3% [9]. Another meta-analysis that included 1084 patients showed a 17.98% decrease in EBWL and 4.4% decrease in TBWL with intragastric balloons, compared to lifestyle interventions alone [11].

Bapaye et al. conducted a meta-analysis on safety and efficacy of adjustable IGBs, demonstrating > 25% EBWL and >10% TBWL in 82.89% of patients, and an even higher success rate of 99.45% after up-adjustment of the IGB. On the other hand, for those patients not tolerating the IGB, 81/125 underwent down-adjustment of the balloon and 72/81 patients subsequently tolerated the IGB with successful weight loss [12]. Similarly, a recently published RCT of 288 patients from seven sites across the US compared adjustable intragastric balloon (aIGB) combined with lifestyle interventions to lifestyle interventions alone. At 32 weeks, TBWL was 15% (95% CI 13.9–16.1) in the aIGB group versus 3.3% (2.0–4.6) in the control group (*p* < 0.0001). Balloon adjustment was required in 80% of patients (intolerance or weight loss plateau), with upward adjustment allowing another 5.2% TBWL. Downward adjustment allowed 75% of patients to complete the full duration of therapy [13].

In 2021 Bazerbachi et al. studied the effect of intragastric balloons on non-alcoholic steatohepatitis in 21 patients with early hepatic fibrosis. EUS-guided liver biopsy and MR elastography (MRE) were done both pre- and 6 months post IGB placement. The authors report an improvement in hepatic fibrosis of 1.5 stages in 50% of patients on MRE, along with an 11.7% TBWL and decrease in HbA1c by 1.3% (*p* = 0.02) [14]. Lee et al. performed a pilot randomized clinical trial of 18 patients in which those with BMI ≥ 27 and histological evidence of NASH were included. The effect of diet, exercise and IGB was compared to diet and exercise alone and studied over 6 months. They demonstrated a change in BMI of 1.5 in the IGB group vs. 0.8 in the control group (*p* = 0.0008), and a decrease in median NAS score of 2 vs. 4 points, respectively (*p* = 0.03). There was no change in histological features of NASH or in fibrosis scores [15].

Common side effects of IGBs include nausea and vomiting (up to 20% of patients) and abdominal pain (about 7% of patients), which may lead to device intolerance and removal. Other less common risks include intestinal bacterial overgrowth, pancreatitis, cholecystitis, gastric ulcer, Mallory Weiss tear related GI bleeding, gastric perforation and even death (0.04%) [9]. Balloon-related complications may include balloon overinflation, spontaneous balloon deflation, migration, impaction or balloon valve malfunction [12]. According to a recent meta-analysis, the total pooled adverse event rate was 10.39% and these were mainly device related AEs [12]. Contraindications to IGB placement include coagulopathy, history of GI tract surgery, GI bleeding and gastric ulcers [9,12,16].

Long-term data on the efficacy of IGB are scant. While presence of IGB for many months may introduce new alimentary habits, and thus more permanent weight loss, it seems that weight regain is frequently encountered in the months/years after IGB removal [17,18,19]. Long-term results are also highly heterogeneous because some patients continue only with lifestyle/diet modifications, some repeat IGB therapy and others receive pharmacological or surgical therapy for obesity. The above studies have been summarized in Table 1.

### 2.2. Endoscopic Sleeve Gastroplasty (ESG)

The idea behind ESG is to be a less invasive alternative to the laparoscopic sleeve gastrectomy (LSG) surgery. The procedure utilizes an endoscopic suturing device (OverStitch; Apollo Endosurgery, Austin, TX) to create a small diameter sleeve (tubular lumen) along the lesser curvature, thereby reducing the volume of the stomach by approximately 70% [20]. The procedure is performed by creating full-thickness endoscopic sutures with different patterns (typically U-, W- or Z-patterns). Different techniques including distal to proximal sutures and reinforcement sutures have helped to decrease gastric volume and increase durability, but no specific suture pattern has been proven to be more efficacious than the others. Suturing of the fundus is generally avoided to allow formation of a “reservoir” of food proximal to the sleeve. This is thought to increase satiety and delay gastric emptying [21,22].

Several meta-analyses have explored the effects of ESG on weight loss and metabolic parameters. In a 2020 systematic review and meta-analysis of 1772 patients, mean TBWL at 6 months was 15.1% (95% CI 14.3–16.0), mean decrease in BMI was 5.65 kg/m^2^ (95% CI 5.07–6.22) and mean EBWL was 57.7 (95% CI 52.0–63.4). Weight loss was sustained at 12 and 18–24 months (TBWL of 16.5%; 95% CI, 15.2–17.8 and 17.2%; 95% CI, 14.6–19.7, respectively). The pooled adverse event rate was 2.2% [23]. Another 2020 meta-analysis found significant pooled TBWL of 8.78% (*p* = 0.00), 11.85% (*p* = 0.00), 14.47% (*p* = 0.02) and 16.09% (*p* = 0.06) at 1, 3, 6 and 12 months, respectively. Similarly, pooled EBWL at 1, 3, 6 and 12 months were 31.16% (*p* < 0.001), 43.61% (*p* < 0.001), 53.41% (*p* < 0.001) and 59.08% (*p* = 0.015), respectively [24]. The recent MERIT trial that compared ESG to diet/lifestyle modifications followed patients for 24 months, with the control group being given the option to cross over at 12 months. The study found an EBWL of 49.2% at 12 months (*p* < 0.0001), with clinically significant reductions in A1c, blood pressure, and lipid levels. At 104 weeks, 68% of patients maintained a >25% EBWL. Serious adverse events were seen in 2% (3 patients), including a peri-gastric abscess, bleeding, and malnutrition. All were successfully treated [25].

In 2021, Hajifathalian et al. followed 118 patients with obesity and NAFLD who underwent ESG, prospectively for two years. Patients were assessed for changes in insulin resistance (measured by the homeostasis model assessment–estimated insulin resistance or HOMA-IR levels) and estimated hepatic fibrosis and steatosis (using the hepatic steatosis index and the NAFLD fibrosis score). Of the 84 patients that completed a 2-year follow up, 24 (20%) had decrease in NAFLD fibrosis score (NFS) from the F3-F4 fibrosis range down to the F0–F2 range. Only one patient had an increase in fibrosis score. HOMA-IR levels improved from 6.7 to 3.01 during the first week after ESG and continued to decrease to 2.0 after the second year of follow up (absolute change per year of −1.7; *p* for trend = 0.02). Similarly, hepatic steatosis index score improved significantly, decreasing by about 4 points per year (*p* for trend < 0.001). Moreover, HbA1c also decreased from baseline of 6.0% to 5.6% at 2 years after ESG, while TBWL and EBWL were 15.5% and 45.5% at the end of the 2-year follow up [26]. In 2021, Jagtap et al. prospectively followed 26 patients who underwent ESG and saw improvements in ALT (−10 iU/L at 6 months and −11 iU/L at 12 months *p* = 0.001), NFS (from 0.228 to −0.202 and −0.52 at 6 and 12 months, respectively, *p* = 0.001), FIB-4 and APRI (aspartate aminotransferase to platelet ratio index) scores at 6 and 12 months, along with significant improvement in HbA1c (−1.5%) and TBWL (18.07% at 12 months) [27]. There are multiple ongoing clinical trials assessing the effect of ESG on NASH and liver fibrosis, using histological data [28,29].

Endoscopic sleeve gastroplasty seems to compare well to traditional laparoscopic sleeve gastrectomy. In a recent case-matched study ESG had a lower TBWL compared to LSG (17.1% ± 6.5% vs. 23.6% ± 7.6%, *p* < 0.01), but also lower adverse event rate (5.2% vs. 16.9%, *p* < 0.05) along with a lower rate of new-onset GERD (1.9% vs. 14.5%, *p* < 0.05) [30]. Similar results were seen in another study comparing 278 patients who had undergone LSG, ESG or laparoscopic gastric banding (LAGB). The overall TBWL was greater in the LSG patients compared to those who underwent LAGB and ESG (29.28% vs. 13.30% vs. 17.57%, respectively, *p* < 0.001). There was a lower adverse event rate in the ESG group compared to LSG and LAGB (2.20 vs. 9.17 vs. 8.96%, *p* value < 0.05). One patient in the ESG group developed a peri-gastric leak. In the LSG group, complications included peri-gastric leak, visceral herniation, pulmonary embolism, prolonged post operative ileus, wound infections, UTI, and significant nausea and vomiting requiring IV fluids. In the LAGB group, complications included gastric outlet obstruction, wound infection, abdominal pain, and pulmonary embolism [31].

Several studies suggest that LSG leads to changes in GLP-1 (Glucagon-like peptide 1) levels as well as changes in bile acid secretion, improves insulin sensitivity, decreases ghrelin, and improves glucose homeostasis [32,33,34]. These hormonal changes are thought to contribute to a higher weight loss observed with LSG compared to ESG, because even though ESG mimics LSG in terms of anatomic alterations it does not seem to reproduce the same hormonal changes [30,35,36]. Hence, some early animal studies suggest that ESG may produce weight loss outcomes similar to LSG if additional therapy, aimed at mimicking the physiologic and hormonal changes seen with LSG, is introduced [37,38]. In a recent propensity-score matched retrospective study, addition of liraglutide (a GLP-1 receptor agonist) to ESG was more effective than ESG alone. Patients who received the additional medical therapy had a higher mean %TBWL at 7 months after initiation of liraglutide compared with those who declined it (24.7% vs. 20.51; *p* < 0.001), as well as greater reduction in percent body fat (7.85% vs. 10.54%; *p* < 0.001) at 12 months [39]. Several ongoing studies are currently evaluating the outcomes of ESG combined with medical therapy.

Contraindications to ESG include large hiatal hernia, bleeding gastric lesions, gastric neoplasm, pregnancy, eating disorders, uncorrectable coagulopathy. Previous bariatric surgery is not an absolute contraindication [40]. The adverse event (AE) rate is about 2%. Potential AEs include nausea, vomiting, abdominal pain, symptomatic GERD, intraprocedural bleeding, subcutaneous emphysema, high end tidal CO2, small amount of pneumoperitoneum from the full thickness sutures and (rarely) tension pneumothorax [40]. These studies have been summarized in Table 2.

### 2.3. Primary Obesity Surgery Endoluminal (POSE)

The POSE procedure uses the Incisionless Operating Platform System (USGI Medical San Clemente, CA, USA) to perform tissue apposition with full thickness plication in the gastric fundus, and thereby reducing gastric volume. Fewer data are available for POSE compared to ESG. The ESSENTIAL Trial was the first multicenter randomized sham-controlled trial for POSE, demonstrating significant TBWL at 12 months of 4.95% in the POSE group vs. 1.38% in the control group [41]. In the subsequent MILEPOST trial, TBWL was seen to be higher at 13% compared to 5.3% in the control group at 12 months [42]. A meta-analysis by Singh et al. included 613 patients undergoing POSE from 7 studies and demonstrated a mean EBWL of 48.86% and TBWL of 12.68% at 12–15 months. In a subgroup analysis of the 2 included RCTs, the POSE procedure group had a 19.45% higher mean EBWL than the control group [43]. A new more distal “belt-and-suspenders” POSE approach has been recently proposed [44].

### 2.4. Endoscopic Gastric Plication (E-ESG)

Gastric Plication is a novel approach using the Endomina TM suturing device, whereby using a triangulation approach, transmural sutures with serosa-to-serosa apposition are placed in the gastric body (anterior to posterior), essentially achieving a gastroplasty. This device is not yet FDA-approved for use in the US. An initial multicenter study of 45 patients showed an EBWL of 29% and TBWL of 7.4% at 12 months, with no severe adverse events [45]. A clinical trial that randomized 21 patients to either E-ESG + lifestyle modification, or lifestyle modification alone, showed an improved EBWL (38.6% vs. 13.4%; *p* < 0.001) and better mean quality of life (52.8% vs. 45.1%, *p* < 0.05) in the E-ESG group at 6 months. At 12 months, EBWL was 45.1%, and TBWL 11.8% [46]. Further studies will be needed to better assess this method.

## 3. Gastric Aspiratoin

### Aspiration Therapy

The AspireAssist device was approved in 2016 for use in patients with BMI 35–55 who have failed lifestyle/medical weight loss interventions. It is placed endoscopically in a similar fashion to a percutaneous endoscopic gastrostomy (PEG) tube and has an attached aspiration catheter that allows for drainage of gastric contents, usually 30 min after a meal. The goal of this device is to physically remove approximately 30% of an ingested meal [47]. The PATHWAY trial compared AspireAssist to lifestyle interventions (82 patients in year 1 and 55 patients beyond year 1) and demonstrated 14.2% TBWL at 1 year, 15.3% at 2 years, 16.6% at 3 years and 18.4% at 4 years. The study also demonstrated decrease in HDL (−8.1 mg/dL at 4 years), serum triglycerides (−31.7 mg/dL at 4 years), HbA1c (−0.034% at 4 years) and ALT levels (−11.9 IU/L at 4 years). The main reasons for discontinuation were fatigue with the therapy or achievement of desired weight loss [48,49]. Adverse effects of AspireAssist are mainly those of PEG tube placement, including peristomal irritation, granulation tissue, infection, nausea, vomiting, abdominal pain, and dyspepsia. Serious adverse effects are rare, including peritonitis and prepyloric ulceration or tube removal [50]. Jirapinyo et al. studied the effect of aspiration therapy on obesity-related comorbidities including NAFLD in 590 patients, and found that at one year there was a significant improvement in systolic blood pressure (−7.8 mm Hg; 95%CI −10.7 to −4.9), diastolic BP (−5.8 mm Hg; 95%CI −7.0 to 3.2), triglycerides (−15.8 mg/dL; 95%CI −24.0 to −7.6), HbA1c (−1.3%; 95%CI −1.8% to −0.8%) and ALT (−7.5 IU/L; 95%CI −9.8 to −5.2), as well as increase in HDL (3.6 mg/dL; 95%CI 0.7 to 6.6). The TBWL was 17.8%. Subgroup analysis of 2 randomized trials demonstrated a pooled SAE rate of 4.1%, with 11.6% TBWL (95% CI 6.5 to 16.7), reduction in HbA1c by 1.3% (95% CI 0.8 to 1.8%), AST −2.7 U/L ((95% CI −4.1 to −1.3), and ALT by 9.0 UL ((95% CI 3.9 to 14.0) [51]. 

Currently this device is not FDA approved, but that seems to be due solely to regulatory issues and not because of patient risk.

## 4. Malabsorptive/Metabolic EBMTs

Currently, most malabsorptive/metabolic EBMTs are undergoing evaluation in clinical trials and are not FDA-approved for routine clinical use.

### 4.1. Duodenal Mucosal Resurfacing (DMR)

Duodenal Mucosal Resurfacing is a minimally invasive endoscopic technique, whereby up to 10 cm of the duodenal mucosa is lifted and then circumferentially hydrothermally ablated, with resultant regeneration of the mucosa [52]. Two devices are currently available, the Revita and DiaGone devices. It has been hypothesized that ablation of the duodenal mucosa may increase insulin sensitivity and achieve weight loss, potentially via increases in unconjugated and secondary bile acid production [53]. In a study with the Revita device there was a drop in HbA1c by 1.2% at 6 months, decrease in Insulin resistance (HOMA-IR) and improvement in fasting glucose levels, as well as weight loss of 2.5 kg. Only 1 patient (2.7%) had a severe adverse event [52]. Interestingly, DMR was shown to achieve good glycemic control independently of effect on BMI [52].

### 4.2. Endoluminal Bypass Liners

It is estimated that 50–70% of insulin secretion is a result of the “incretin effect”, i.e., release of incretins following food intake. This mechanism appears to be downregulated in patients with diabetes or obesity. The two main incretins are glucagon-like-peptide 1 (GLP-1) and glucagon dependent insulinotropic polypeptide (GIP), which are familiar antidiabetic drug targets [54]. The Endoluminal bypass liner (Endobarrier; GI Dynamics, USA) has been introduced as a method to increase the incretin effect. It works by providing a physical barrier to the proximal duodenal mucosa, thus decreasing interaction of chyme with the duodenal mucosa. The consequence is that there is a decrease in the production of anti-incretins, which leads to increased incretin production, increased bile acid production as possibly favorable modifications of the gut microbiota [54,55]. The Endobarrier device remains in place in the duodenal bulb for 3–12 months, requiring subsequent endoscopic removal. In a recent study of 90 patients treated with Endobarrier, after one year there was a mean 15.9 kg TBWL (*p* < 0.001), BMI decrease of 5.7 kg/m^2^ (*p* < 0.001), 3.9% decrease in HbA1c (*p* < 0.001), as well as significant decreases in systolic blood pressure, serum cholesterol, ALT levels. One year following Endobarrier removal, 45% of patients fully sustained improvement in weight loss and glycemic control, 35% of patients sustained some improvement, but not completely, and 20% of patients went back to baseline weight and diabetes control. Early removal occurred in 13/90 patients: five for GI bleeding, two for liver abscess and one for intraabdominal abscess [56]. A recent meta-analysis of five RCTs (235 subjects) and 10 observational studies (211 subjects) showed an overall EBWL of 12.6% (95% CI 9.0 to 16.2) compared to diet modification alone, with a mean weight loss of 5.1 kg. The authors also found a drop in HbA1c (−0.9%) and fasting glucose levels (−3.7 mm), but neither of these values reached statistical significance [55]. 

Gollisch et al. studied the effect of the Endobarrier on liver fibrosis (measured by liver elastography). A total of 13/19 patients had F2 fibrosis at the time of endobarrier implantation, with reduction to a normal liver stiffness at the time of endobarrier explantation at one year (*p* < 0.01). Steatosis measurements (CAP score via elastography) did not show a significant change in steatosis grade [57].

Adverse events included abdominal pain, nausea and vomiting [55]. An earlier version of the Endobarrier was pulled off the market in 2015, after the ENDO trial was halted early due to development of seven liver abscess, with post market analysis also suggesting an increased risk of liver abscess development. This was hypothesized to be due to the liner becoming a focus of infection with contiguous spread to the liver bed. At this time, the EndoBarrier is only licensed to remain in situ for one year for this reason [54].

Hoffmeister et al. reported their findings from the weight loss endoscopy (WET) double blinded, sham controlled trial, comparing weight loss achieved with implantation of duodenojejnual bypass liner (DJBL) vs. intragastric balloon vs. sham procedure group (2:2:1 randomization). The study was terminated early due the duodenojejunal bypass liner being pulled from the market, as above. The primary outcome was sustained weight loss > 10% from baseline at 12 months following device explanation. All patients achieved weight loss while the device remained implanted (DJBL: 129.4 ± 28.3 kg to 107.4 ± 16.7 kg; IGD: 118.3 ± 22.8 kg to 107.4 ± 25.7 kg; sham: 134.6 ± 18.0 kg to 131.2 ± 14.3 kg at 12 months. However, following device explanation only one patient in the intragastric balloon group maintained > 10% TBWL [58]. Another sham controlled trial by Gersin et al. of the DJBL assessed the difference in EBWL after 12 weeks in 13 patients who underwent DJBL implantation, compared to 24 patients in the sham group. EBWL was 11.9% ± 1.4% in the DJBL arm compared to 2.7% ± 2.0% in the sham arm (*p* < 0.05). TBWL was −8.2 ± 1.3 in the DJBL group compared with −2.1 ± 1.1 kg in the sham group (*p* < 0.05). Side effects were GI bleeding, abdominal pain, nausea and vomiting [59].

### 4.3. Endoscopic Anastomosis Devices 

The Incisionless Magnetic Anastomosis System (IMAS) entails placement of magnets in adjacent bowel loops to create a non-surgical jejuno-jejunal anastomosis, and therefore creating a partial jejunal diversion. The IMAS magnets are delivered via a colonoscope with laparoscopic supervision. At the first in-human pilot study this technique showed TBWL of 14.6% and EBWL of 40.2% at 12 months. Significant HbA1c reduction of 1.9% was also seen over this time period [60].

These studies on EBMTs other than IGB and ESG have been summarized in Table 3.

## 5. Overall Effects of EBMTs for Non-Alcoholic Fatty Liver Disease

While there have been studies that have evaluated each of the modalities above with respect to the effects they have on NAFLD, two recent meta-analyses have evaluated the global effect of EBMTs on this disease.

The first is a meta-analysis that included 863 patients in 18 studies. The study found an average TBWL of 14.5% at 6 months (95%CI 12.9 to 16.2, *p* < 0.001), with reduction in liver fibrosis by standardized mean difference (SMD) of 0.7 (95% CI 0.1–1.3, *p* = 0.02). There was significant reduction in ALT of −9.0 U/L (95% CI −11.6 to −6.4, *p* < 0.0001), hepatic steatosis SMD −1.0 (95% CI −3.5 to −1.5; *p* < 0.0001), NAFLD Activity score by −2.5 (95% CI −3.5 to −1.5, *p* < 0.001), decrease in waist circumference by −4.5 inches (95% CI −5.5 to −4.2, *p* < 0.001), decrease in HOMA- IR by −1.8 (95% CI −2.5 to −1.2, *p* < 0.001) and reduction in HbA1c by −0.2 (95% CI −0.4 to −0.1, *p* = 0.02). Most of these studies utilized intragastric balloons: two studies used ESG and two studies used aspiration therapy [5].

Another recent meta-analysis included 1710 patients in 33 studies. Although there was significant heterogeneity in the method of liver fibrosis assessment, the authors found a significant decrease in NAFLD fibrosis score with mean difference −0.58 (95% CI −0.97 to −0.20), and a non-statistically significant decrease in TE (transient elastography) detected liver stiffness of −6.39 kPa (95% CI −13.73 to 0.96, n = 91), FIB-4 by −0.028 (95% CI −0.63 to 0.07, n = 49). One study using IGBs did significantly reduce aspartate aminotransferase to platelet ratio index (APRI) by 0.73 (*p* = 0.005) and MRE detected liver stiffness by 0.3 kPa (*p* = 0.03). Significant reduction in hepatic steatosis (mean decrease in CAP score −53.76 dB/m [95% CI −73.04 to −34.47), NAFLD activity score (evaluated in 2 studies, mean decrease −3 [95% CI −3.27 to −2.73]) and HOMA-IR levels were seen, as well as decrease in ALT (mean decrease −12.44 U/L [95% CI −14.70 to −10.19]), AST (mean decrease −7.88 U/L [95% CI −11.11 to −4.64]), GGT (−12.07 U/L [95% CI −15.79 to −8.35]), triglyceride, total cholesterol levels and total body weight. The most common EBMT modalities that were used included IGBs in 19 studies, DMR = 3 studies, endobarrier in 8 studies, ESG in 3 studies, POSE in 1 study and aspiration therapy in 2 studies [4].

## 6. Comparison of Weight Loss with Different Types of EBMTs

There have been very few studies comparing the efficacy of different types of endoscopic bariatric therapies. Two recent studies compared weight loss with intragastric balloons versus with endoscopic sleeve gastroplasty and showed higher TBWL at 6 and 12 months with ESG. Kozlowska-Petriczko et al. demonstrated TBWL of 19.8% for ESG vs. 15.3% IGB at 6 months (*p* = 0.005). TBWL at 12 months was 22.5% for ESG vs. 14.7% for IGB (*p* < 0.001). Similarly, Fayad et al. demonstrated TBWL of IGB vs. ESG at 1 month: 6.6% [2.6%] vs. 9.9% [2.4%]; *p* < 0.001, at 3 months: 11.1% [4.4%] vs. 14.3% [4.6%]; *p* = 0.004, at 6 months: 15.0% [7.6%] vs. 19.5% [5.7%]; *p* = 0.01, and at 12 months: (13.9% [9.0%] vs. 21.3% [6.6%]; *p* = 0.005). The effect of IGB tended to wane after balloon removal at 6 months [61,62]. Jung et al. compared the TBWL and EBWL achieved with gastric aspiration, IGB, POSE and to DJBL, and showed that the highest weight loss was achieved with Gastric Aspiration(TBWL 10.4% [7.0% to 13.7%]), followed by IGB (5.3% [3.4% to 7.2%]), then POSE (4.9% [1.7% to 8.2%] and DJBL (4.5% [1.4% to 7.7%]) [63]. The results of these studies have been summarized in Table 4.

## 7. Conclusions

While the advent of endoscopic bariatric and metabolic therapies is relatively recent, there is already a large body of literature suggesting that these procedures are safe and effective non-surgical, minimally invasive options for patients unable/unwilling to undergo surgery for weight loss and management of obesity-related comorbidities. Currently, most available evidence is about intragastric balloons and endoscopic sleeve gastroplasty. However, more evidence is being accumulated regarding other methods, and new devices are being developed or adapted.

The available evidence on the long-term effects of EBMTs is relatively scant with no long-term survival data available at this time. More studies will be needed to identify the best subpopulation of patients that may benefit from EBMTs rather than, or in combination with, other approaches. While more studies will be needed to define their proper role, it seems clear that EBMTs should be part of the standard options offered to patients in any institution with a bariatric program.

## Figures and Tables

**Table 1:** Weight loss with Intra-gastric Balloons.

Author (Year)	Study Design	EBMT Type (n)	Outcome and Assessment Timeframe	Weight Loss Achieved	Changes in Metabolic Parameters
Dayyeh (2021) [13]	RCT	a-IGB vs. control (n = 288)	Weight loss at 32 weeks	TBWL: 15.0% (13.9–16.1) in a-IGB group vs. 3.3% (2.0–4.6) in control group	Δ HbA1c:–0.73% (95% CI –1.49 to 0.02; *p* = 0.055) Total cholesterol:–6.8 mg/dL (–11.1 to –2.6; *p* = 0.0018) SBP:–6.1 mm Hg (–9.8 to –2.3; *p* = 0.0016) DBP: –3.7 mm Hg (–6.4 to –1.0; *p* = 0.0078) Significant decrease in ALT and AST
Popov (2017) [9]	Meta-Analysis	IGB (n = 5688)	Changes in metabolic parameters (time period unspecified)	EBWL: 28% (23.5 to 32) Δ BMI: −4.8 kg/m^2^ (−6.3 to −3.3)	FPG: −12.7 mg/dL (−21.5, −4) Δ HbA1c: −1.1% (−1.6 to −0.6) TG: −19 mg/dL (−4 to 3.5) Waist circumference: −4.1 cm (−6.9 to −1.4) DBP: −2.9 mmHG (−4.1 to −1.8) AST: −3 U/L (−5.6 to −0.1) ALT: −9 U/L (−12 to −5.2)
Kotinda (2020) [11]	Meta-Analysis	IGB (n = 1523)	Weight loss at 3–8 months	Δ TBWL between IGB and sham groups: 4.40% (1.37–7.43) Δ EBWL between IGB and sham groups: 17.98% (95%CI 8.37–27.58)	NA
Bapaye (2022) [12]	Meta-Analysis	a-IGB (n = 866)	Weight loss	>10% TBWL and >25% EBWL in 82.89% patients (80.82–85.52%)	NA
Dayyeh (2015) [7]	Meta-Analysis	IGB (n = 1683)	Weight loss at 12 months	EBWL at 12 months: 25.44% (21.47–29.41%) TBWL at 12 months: 11.27% (8.17–14.36%)	NA
Bazerbachi (2014) [14]	Open label, prospective study	IGB (n = 21)	Changes in liver histology parameters, weight and metabolic parameters at 6 months	TBWL: 11.7% ± 7.7%	Δ HbA1c (1.3% ± 0.5%) (*p* = 0.02) Δ waist circumference: 14.4 ± 2.2 cm (*p* = 0.001) Median Δ NAS: 3 points(range 1–4) MRE detected fibrosis: improvement by 1.5 stages (50% patients)
Lee (2012) [15]	RCT	IGB vs. sham (n = 18)	Change in liver histology after 6 months	Δ BMI: −1.52 IGB group vs. −0.8 sham group (*p* = 0.0008)	NAS score: 2 [0.75] in IGB group vs. 4 [2.25] in sham group; (*p* = 0.03)

IGB: Intragastric balloon, a-IGB: adjustable Intragastric balloon; NA: Not Assessed; RCT: randomized controlled trial; CAP score = controlled attenuation parameter as a measurement of steatosis on liver elastography; HbA1c: glycosylated hemoglobin; FPG: fasting plasma glucose; HOMA-IR: Homeostatic Model assessment of Insulin Resistance; SBP Systolic Blood pressure; DBP: Diastolic Blood Pressure; TG: Triglycerides; HDL: High density lipoprotein Cholesterol; LDL: Low Density Lipoprotein cholesterol; AST: Aspartate aminotransferase; ALT: Alanine Transaminase; NFS: NAFLD Fibrosis Score; FIB-4: Fibrosis-4-index; APRI: AST-to-platelet ratio index; HIS: hepatic steatosis index; NAS score: Nonalcoholic fatty liver disease activity score; DM: diabetes mellitus; HTN: Hypertension; HLD: hyperlipidemia; TBWL: Total Body Weight loss; EBWL: excess Body weight loss; Δ: change in.

**Table 2:** Weight loss with Endoscopic Sleeve Gastroplasty.

Author (Year)	Study Design	EBMT Type (n)	Outcome and Assessment Timeframe	Weight Loss Achieved	Changes in Metabolic Parameters
Hedjoudje (2020) [23]	Meta-Analysis	ESG (n = 1772)	Weight loss at 6, 12 and 18–24 months.	TBWL: 6 months: 15.1% (14.3–16.0) 12 months: 16.5% (15.2–17.8) 18–24 months: 17.2% (14.6 −19.7) EBWL at 6 months: 57.7% (52.0–63.4)	NA
Li (2020) [24]	Meta-Analysis	ESG (n = 1542)	Weight loss 1, 3, 6 and 12 months.	TBWL: 1 month:8.78% 3 months: 11.85% 6 months:14.47% 12 months: 16.09% EBWL: 1 month: 31.16% 3 months: 43.61% 6 months:53.14% 12 months: 59.08%	NA
Dayyeh (2022) [25]	RCT	ESG vs. control (n = 209)	Weight loss and changes in metabolic comorbidities by 52 weeks.	EBWL: 49.2% ESG vs. 3.2% control group (*p* < 0.0001) TBWL: 13.6% for ESG group vs. 0.8% for control (*p* < 0.0001)	Improvement in DM, HTN, HLD, metabolic syndrome (numbers not provided)
Hajifthalian (2021) [26]	Prospective Cohort	ESG (n = 118)	change in IR and estimated hepatic steatosis and fibrosis 2 yrs after ESG.	TBWL at 2 yrs: 15.5% (13.3–17.8%). EBWL at 2 yrs: 45.5%(38.1–52.8%)	2 yrs: HOMA-IR: −1.7 per year HbA1c: −0.4% HSI: −4 points/year NFS: −0.3 points/year ALT: −5 U/L/year AST −3 U/L/year Leptin: −3.8 ng/mL/year
Jagtap (2021) [27]	Prospective Cohort	ESG (n = 26)	Impact of ESG on hepatic parameters, metabolic parameters at 6 and 12 months.	TBWL: 18.07% at 12 months	ALT (mean ± SD) from baseline: 59.54 ± 17.02 IU/L to 49.50 ± 11.72 IU/L and 48.42 ± 13.22 IU/L at 6 and 12 months (*p* = 0.001). Mean (SD) NFS: 0.228 (1.00) at baseline to −0.202 (1.16) and −0.552 (1.08) at 6 and 12 months (*p* = 0.001).
Fayad (2019) [30]	Retrospective Case control	ESG (n = 54) vs. LSG (n = 83)	Weight loss at 6 months.	TBWL: 17.1% ± 6.5% with ESG vs. 23.6% ± 7.6% with LSG *p* < 0.01	NA
Novikov (2018) [31]	RCT	ESG vs. LSG vs. LAGB (n = 278)	Weight loss at 3, 6, 9 and 12 months.	TBWL: LSG vs. LAGB vs. ESG: 29.28 vs. 13.30 vs. 17.57%, respectively; *p* < 0.001)	NA

ESG: Endoscopic Sleeve Gastroplasty, LSG: Laproscopic Sleeve gastrectomy; LAGB: Laproscopic adjustable gastric banding; NA: Not Assessed; RCT: randomized controlled trial; CAP score = controlled attenuation parameter as a measurement of steatosis on liver elastography; HbA1c: glycosylated hemoglobin; FPG: fasting plasma glucose; HOMA-IR: Homeostatic Model assessment of Insulin Resistance; SBP Systolic Blood pressure; DBP: Diastolic Blood Pressure; TG: Triglycerides; HDL: High density lipoprotein Cholesterol; LDL: Low Density Lipoprotein cholesterol; AST: Aspartate aminotransferase; ALT: Alanine Transaminase; NFS: NAFLD Fibrosis Score; FIB-4: Fibrosis-4-index; APRI: AST-to-platelet ratio index; HIS: hepatic steatosis index; NAS score: Nonalcoholic fatty liver disease activity score; DM: diabetes mellitus; HTN: Hypertension; HLD: hyperlipidemia; TBWL: Total Body Weight loss; EBWL: excess Body weight loss.

**Table 3:** Weight loss with other EBMTs.

Author (Year)	Study Design	EBMT Type (n)	Outcome and Assessment Timeframe	Weight Loss Achieved	Changes in Metabolic Parameters
Sullivan (2017) [41]	RCT	POSE (n = 332)	Weight loss at 12 months, changes in metabolic conditions at 12 months.	TBWL: 4.95 ± 7.04% in active group vs. 1.38 ± 5.58% in the sham group *p* < 0.0001	SBP: −4.78 mm HG DBP: 2.92 mm Hg FPG: −2.18 mmol/dL HbA1c: −0.07% Total cholesterol: −7.07% HDL: +3.15 LDL: −6.81 TG:−11.5
Miller (2017) [42]	RCT	POSE (n = 44) vs. control	Weight loss at 12 months	TBWL:13.0% vs. 5.0% in control group EBWL: 45.0% vs. 18.1% in control group. (*p* < 0.01)	NA
Singh (2021) [43]	Meta Analysis	POSE (n = 613)	Weight loss at 3–6 months and 12–15 months.	EBWL: 3–6 mo: 42.62% (95% CI 37.56–47.68) 12–15 mo: 48.86% (95% CI 42.31–55.41) TBWL: 3–6 mo:13.45% (95% CI 8.93–17.97) 12–15 mo: 12.68% (95% CI 8.13–17.23)	NA
Huberty (2018) [45]	RCT	E-ESG (n = 51) vs. control	Weight loss at 1 yr.	EBWL: 29% TBWL: 7.4%	NA
Huberty (2021) [46]	RCT	E-ESG (n = 71) vs. control	EBWL >25% by 12 months, ≥15% EBWL difference between groups at 6 months	6 months: EBWL: 38.6% vs. 13.4%, *p* < 0.001 12 months EBWL = 45.1% TBWL = 11.8%	NA
Thompson (2019) [49]	RCT	Gastric Aspiration Therapy (n = 81) vs. control	Weight loss at years 1, 2, 3 and 4.	1 yr (n = 82): TBWL = 14.2%, EBWL = 37.1% 2 yr (n = 42): TBWL = 15.3%, EBWL = 40.8% 3 yr (n = 22): TBWL = 16.6%, EBWL = 44.7% 4 yr (n = 15): TBWL = 18.7%, EBWL = 50.8% (*p* < 0.01 for all)	4 y results: SBP: −10.5 ± 16.2 mmHg DBP: −2.1 ± 13.6 mmHg HDL: +7.7 ± 9.2 mg/dL LDL: 2.0 ± 23.2 mg/dL TG: −38.6 ± 74.1 mg/dL HbA1c: −0.33 ± 0.6% ALT: −11.9 ± 11.5 IU/L AST: −6.1 ± 6.6 IU/L
Thompson (2017) [50]	RCT	Gastric Aspiration Therapy compared to controls (n = 207)	Mean EBWL at 52 weeks	Aspiration Therapy group: TBWL: 12.1 ± 9.6% EBWL: 31.5 ± 26.7% Control group: TBWL: 3.5 ± 6.0% EBWL 9.8 ± 15.5% (*p* < 0.001)	HbA1C:−0.36% relative to 5.7% baseline, *p* < 0.0001, TG: −9.9%, *p* = 0.02 HDL: +8.1%, *p* = 0.0001 SBP:−1.2%, *p* = 0.38 DBP: −2.6%, *p* = 0.06 LDL:−4.2%, *p* = 0.06 Total cholesterol: −2.5%, *p* = 0.07
Jirapinyo (2020) [51]	Meta-Analysis	Gastric Aspiration Therapy (n = 590)	Changes in metabolic comorbidities at 1 yr, weight loss up to 4 yrs.	1 yr (n = 218) TBWL: 17.8% EBWL: 46.3% 2 yrs (n = 125) TBWL: 18.3% EBWL: 46.2% 3 yrs (n = 46) TBWL: 19.1% EBWL: 48.0% 4 yrs (n = 27) TBWL: 18.6% EBWL: 48.7% (*p* <0.0001 for all)	1 yr: SBP: −7.8 (−10.7–−4.9) mm Hg DBP −5.1 (−7.0–3.2) mm Hg TG:−15.8 (−24.0–−7.6) mg/dL; HDL: 3.6 (0.7–6.6) mg/dL HbA1c: −1.3 (−1.8–−0.8)% AST: −2.7 (−4.1–−1.3) U/L ALT: −7.5 (−9.8–−5.2) U/L
Van Baar (2020) [52]	Prospective, open label, multicenter study	DMR (n = 46)	Effect on glucose levels at 24 weeks and 12 months	TBWL at 24 weeks: −2.5 ± 0.6 kg (*p* < 0.001)	24 Weeks HbA1c: −10 ± 2 mmol/mol (−0.9% ± 0.2%) * FPG: −1.7 ± 0.5 mmol/L * HOMA-IR: −2.9 ± 1.1 * 12 months: HbA1c:−10 ± 2 mmol/mol (−0.9% ± 0.2%) * FPG: −1.8 ± 0.5 mmol/L (*p* < 0.001) HOMA-IR: 3.3 ± 0.9 * * *p* < 0.001 for all values
Gollisch (2017) [57]	Retrospective Cohort study	EndoBarrier (n = 19)	Change in liver fibrosis by 12 months	NA	Δ Liver elastography measurements: Fibrosis score: from 10.4 kPa (IQR 6.0–14.3) at baseline to 5.3 kPa (IQR 4.3–7.7, *p* < 0.01) CAP score: from 343 dB/m (IQR 326–384) to 317 dB/m (IQR 269–375, *p* < 0.05)
Hoffmeister (2022) [58]	RCT	DJBL(n = 11) vs. IGB(n = 15) vs. sham (n = 7)	Weight loss by 12 months	TBWL: DJBL:129.4 ± 28.3 to 107.4 ± 16.7 kg IGB:118.3 ± 22.8 to 107.4 ± 25.7 kg sham:134.6 ± 18.0 to 131.2 ± 14.3 kg	NA
Gersin (2010) [59]	Prospective, sham controlled RCT	DJBL (n = 13 DJBL vs. 24 in sham arm)	Weight loss by 12 weeks	TBWL: −8.2 ± 1.3 kg in DJBL vs. −2.1 ± 1.1 kg in sham (*p* < 0.05) EBWL: 11.9 ± 1.4% DJBL vs. 2.7 ± 2.0% in sham (*p* < 0.05)	NA
Machytka (2017) [60]	Pilot RCT	IMAS (n = 10)	Weight loss by 12 months	TBWL = 14.6% EBWL = 40.2%	ΔHbA1c −1.9% (diabetic patients) and −1.0% in prediabetic patients.

POSE: Primary Obesity Surgery Endoluminal; E-ESG: Endoscopic Gastric Plication; DMR: Duodenal Mucosal resurfacing; DJBL: Duodenojejunal Bypass Liner; IMAS: Incisionless Magnetic Anastomosis System, NA: Not Assessed; RCT: randomized controlled trial; CAP score = controlled attenuation parameter as a measurement of steatosis on liver elastography; HbA1c: glycosylated hemoglobin; FPG: fasting plasma glucose; HOMA-IR: Homeostatic Model assessment of Insulin Resistance; SBP Systolic Blood pressure; DBP: Diastolic Blood Pressure; TG: Triglycerides; HDL: High density lipoprotein Cholesterol; LDL: Low Density Lipoprotein cholesterol; AST: Aspartate aminotransferase; ALT: Alanine Transaminase; NFS: NAFLD Fibrosis Score; FIB-4: Fibrosis-4-index; APRI: AST-to-platelet ratio index; HIS: hepatic steatosis index; NAS score: Nonalcoholic fatty liver disease activity score; DM: diabetes mellitus; HTN: Hypertension; HLD: hyperlipidemia; TBWL: Total Body Weight loss; EBWL: excess Body weight loss; Δ: change in. * *p* < 0.001 for all values.

**Table 4:** Comparison of EBMTS.

Author (Year)	EBMTS Compared	Weight Loss	Adverse Events
Fayad (2019) [61]	IGB (n = 58) vs. ESG (n = 47)	TBWL% WITH IGB vs. ESG 1 month (6.6% [2.6%] vs. 9.9% [2.4%]; *p* < 0.001) 3 months (11.1% [4.4%] vs. 14.3% [4.6%]; *p* = 0.004) 6 months (15.0% [7.6%] vs. 19.5% [5.7%]; *p* = 0.01), 12 months (13.9% [9.0%] vs. 21.3% [6.6%]; *p* = 0.005).	IGB group = 17% vs. ESG group = 5.2% (*p* = 0.048).
Jung (2020) [63]	Gastric aspiration vs. IGB vs. POSE vs. DJBL compared to controls	TBWL % Gastric Aspiration = 10.4% [7.0% to 13.7%] IGB = 5.3% [3.4% to 7.2%] POSE 4.9% [1.7% to 8.2%] DJBL 4.5% [1.4% to 7.7%] EBWL % Gastric aspiration = 27.3% [15.3% to 39.3%] IGB = 22.4% [15.4% to 29.4%] POSE = 15.3% [2.5% to 28.0%] DJBL = 13.0% [4.9% to 21.2]	Overall AEs per group not assessed
Kozlowska-Petriczko (2022) [62]	ESG vs. IGB	TBWL% ESG vs. IGB 6 months: 19.8% vs. 15.3% (*p* = 0.005) 12 months: 22.5% vs. 14.7% (*p* < 0.001)	IGB removal due to intolerance in 10.7% patients. ESG AEs not reported.

IGB: Intragastric balloon, ESG: Endoscopic Sleeve Gastroplasty, POSE: Primary Obesity Surgery Endoluminal; DMR: Duodenal Mucosal resurfacing; DJBL: Duodenojejunal Bypass Line; NA: Not Assessed; TBWL: Total Body Weight loss; EBWL: excess Body weight loss.

## Data Availability

Not applicable.
